# The Flipped Journal Club

**DOI:** 10.5811/westjem.2017.11.34465

**Published:** 2017-12-22

**Authors:** Richard Bounds, Stephen Boone

**Affiliations:** * Christiana Care Health System, Department of Emergency Medicine, Newark, Delaware; † University of Vermont Medical Center, Burlington, Vermont; ‡ Baylor College of Medicine, Department of Emergency Medicine, Houston, Texas; § Baylor College of Medicine, Department of Internal Medicine, Houston, Texas

## Abstract

**Introduction:**

Educators struggle to develop a journal club format that promotes active participation from all levels of trainees. The explosion of social media compels residencies to incorporate the evaluation and application of these resources into evidence-based practice. We sought to design an innovative “flipped journal club” to achieve greater effectiveness in meeting goals and objectives among residents and faculty.

**Methods:**

Each journal club is focused on a specific clinical question based on a landmark article, a background article, and a podcast or blog post. With the “flipped” model, residents are assigned to prepare an in-depth discussion of one of these works based on their level of training. At journal club, trainees break into small groups and discuss their assigned readings with faculty facilitation. Following the small-group discussions, all participants convene to summarize key points. In redesigning our journal club, we sought to achieve specific educational outcomes, and improve participant engagement and overall impressions.

**Results:**

Sixty-one residents at our emergency medicine program participated in the flipped journal club during the 2015–2016 academic year, with supervision by core faculty. Program evaluation for the flipped journal club was performed using an anonymous survey, with response rates of 70% and 56% for residents and faculty, respectively. Overall, 95% of resident respondents and 100% of faculty respondents preferred the flipped format.

**Conclusion:**

The “flipped journal club” hinges upon well-selected articles, incorporation of social media, and small-group discussions. This format engages all residents, holds learners accountable, and encourages greater participation among residents and faculty.

## INTRODUCTION

Journal club is an essential component of graduate medical education, used to teach trainees how to critically appraise the literature and integrate evidence-based medicine into practice.^1^–^3^ However, many challenges are encountered when designing an effective journal club structure that actively engages learners and accomplishes these objectives.^4^,^5^,^6^ Most programs assign designated residents to deliver an oral presentation,^7^ while the remainder of the participants have little motivation to prepare beforehand and risk becoming passive listeners. The traditional Socratic method of “calling on” residents may encourage participation, but may also conflict with the desire to create a collegial atmosphere. Another challenge is generating discussion that is appropriate to all levels of training.

At our emergency medicine (EM) residency program, we sought input from residents and faculty to identify strengths and weaknesses of our traditional format in order to prioritize goals and objectives for journal club. We then designed a “flipped journal club” to implement for one academic year prior to program evaluation.

## METHODS

### Setting

Our residency consists of a postgraduate-year (PGY) 1–3 EM program, as well as two five-year combined training programs in EM/IM and EM/FM, for a total of 61 residents. Our curriculum includes a monthly journal club held at a restaurant, conference center, or faculty member’s home. All residents on EM rotations are required to attend.

### Traditional journal club format

Our residents traditionally were assigned in groups of three to lead a journal club. This group would select articles based on the curriculum topic for that block, with approval from a faculty member, and distribute the articles to all participants. On the evening of journal club, the designated residents would each present their chosen article. This was followed by an open discussion among residents and faculty.

Feedback from residents and faculty (through discussions after journal club, unsolicited emails, quarterly class meetings, and Curriculum Committee and Program Evaluation Committee meetings) indicated numerous drawbacks with this format. Some residents were nervous about speaking in front of a large audience. Much of the audience was ill-prepared and disengaged, and the ensuing discussions were generally led by a handful of extroverted residents. Furthermore, the relevance and quality of selected articles was inconsistent. Lastly, we found that residents were using various social media sources rather than reading original research.

### Flipped journal club

We developed a new journal club format to effectively engage all learners using three methods: a focus on specific topics associated with landmark articles, incorporation of social media resources, and division into small groups for discussion. These changes were reflective of flipped-classroom models being integrated into other parts of the curriculum.

We designated a senior faculty member (an associate program director [APD]) and a chief resident as a leadership team to champion the redesigned format. Each month, they select an important controversial clinical topic with an associated “landmark article.” A group of three residents, comprised of a PGY-1, PGY-2, and PGY-3, 4, or 5, are selected by the chief resident based on schedule availability. The resident group reviews the landmark article and selects an accompanying background article and a social media piece that is relevant to the topic and freely accessible to the residents. These selections require approval by the leadership team.

The articles and the social media selection are distributed to all residents and faculty one week prior to each journal club. The three residents must additionally prepare a “facilitator’s guide” with summaries and discussion points (Appendix A). The leadership team reviews this document prior to distribution to faculty small-group facilitators.

On the evening of journal club, all residents are divided into small groups (typically 5–8 members), composed of learners from all levels of training and facilitated by a faculty member. Within the small groups, interns are expected to discuss the background article. The PGY-2 residents present their analysis of the landmark article, followed by the senior residents’ critique of the social media piece. Residents truly lead the discussion, while the faculty facilitators pose questions and provide oversight. Following the small-group discussions, everyone reconvenes to openly summarize and debate key points.

Following each session, the three designated residents create a summary of the main discussion points to be electronically distributed to all faculty and residents (Appendix B).

### Program Evaluation

In redesigning our journal club, we sought to achieve specific educational outcomes, and improve participant engagement and overall impressions. We initiated the flipped journal club in July 2015, and following one full academic year we evaluated our educational outcome measures using an anonymous online survey (Appendix C). The voluntary survey, previously pilot tested in a sample of the target population, was sent to all EM residents (n=61) and core academic faculty (n=16). To provide a framework for evaluation, participants were asked to first select their personal goals for journal club from a comprehensive list based on prior literature,^1^,^2^,^8^ and then choose which format most effectively met the educational objectives.

The Christiana Care Institutional Review Board determined that the program evaluation survey was exempt under an educational curricula waiver.

## RESULTS

Of the 61 residents who received the survey, 43 responded (70%). Ten were interns and, thus, unable to compare formats. Of the 16 faculty, nine responded (56%), with all having experienced both formats. Four of the seven faculty who did not respond attended journal club only once or not at all during the study period.

We first asked participants to select what they hoped to gain from journal club from a list of options^1^,^9^ (Table 1). Next, we asked respondents to choose which format best facilitated learning objectives, and which was preferred in terms of overall impression. Interns who were unable to compare the two formats were excluded, leaving 33 residents for analysis. Overall, the flipped journal club format was preferred over the traditional format for every domain and there was no difference in preference between faculty and residents (all p-values > 0.05). (see table 2)

Over 90% of the residents reported that they more often arrive prepared for the new format, and greater than 95% responded that the flipped journal club better allowed them to contribute to the discussion. The final question of the survey asked residents if we should continue with the flipped journal club and 95% responded positively. All faculty respondents felt that we should continue to use the new format.

## DISCUSSION

In the ever-changing landscape of medical education, innovative techniques for engaging learners of all levels of training must continue to be developed and refined. Our innovation uses small groups, focused clinical topics with a related landmark article, and medical education through social media. This format was preferred by both trainees and faculty in regard to personal goals, educational objectives, and overall impressions. The changes to our format reflect the concepts of the “flipped learning” model in which the four pillars include: a flexible environment, learning culture, intentional content, and a professional educator.^10^

The small-group format allows *flexibility* in the pace and the focus of the discussion and increases the level of active participation. In this *learner-centered* setting, residents drive the discussion and stimulate insightful conversations that create opportunities for more senior participants to teach and share experiences. An experienced team *intentionally* chooses impactful articles that merit in-depth review. The works selected by the designated resident teams are vetted to verify relevance and accessibility. Previous literature indicates residents endorse open-access medical education as their most beneficial source of education, yet also reports infrequent review of the references or quality of evidence.^11^ The Council of Residency Directors in Emergency Medicine acknowledges the valuable role of social media in enhancing education and recommends that programs integrate social media into curricula.^12^ Faculty have a responsibility to help residents sift through this vast resource.^13^ Our “flipped journal club” allows *professional educators* the opportunity to fulfill this duty.

There are potential barriers to implementation of this format. Faculty may need training in small-group facilitation. Continued leadership is necessary to maintain high standards in article selection, review social media resources, and hold residents accountable. In our experience, the designated chief resident and faculty spent 1.5–2 hours per month reviewing articles and coordinating with the assigned teams, generally via email. Our program assigns one chief resident to assist with organization and implementation of our educational curriculum, and journal club leadership naturally fits into that individual’s roles and responsibilities. This structure may not be in place at all programs.

This innovation may be feasible for other EM programs, as well as other medical specialties. The similarities to the flipped-classroom model used in conference formats will be familiar to most educators. Some programs may also consider adopting individual components of this format to augment their current journal club. Future research may include evaluation of the feasibility of this model in other programs and studying the effectiveness of each individual intervention (such as a pre-post effectiveness study).

## LIMITATIONS

Study limitations include a small sample size of participants from a single, large EM residency program as well as a relatively low response rate to the survey, which has potential to bias the results. Validity evidence was not collected for the survey. Potential confounders inherent to the observational nature of our study include changes in core faculty and residents from one year to the next. Furthermore, the study was not designed to assess the impact of the individual components of the flipped journal club model. Lastly, the measured response to our intervention was limited to a post-implementation survey of self-reported goals and objectives obtained after the study intervention.

## CONCLUSION

The “flipped journal club” hinges upon well-selected articles, incorporation of social media, and small-group discussions. The format is meant to promote accountability and create an atmosphere that encourages dialogue among all participants. In our study, the modifications to journal club improved the sense of achieving both personal goals and targeted educational objectives, and was strongly favored by residents and faculty.

## Supplementary Information







## Figures and Tables

**Table 1:** Personal goals for journal club (respondents could select more than one). Total number of respondents was 33 for residents and 8 for faculty.

Personal goals for journal club (listed in order of resident responses)	% (#) Selected by residents	% (#) Selected by faculty
Improve my knowledge of current EM literature	80% (33)	100% (8)
Learn from my colleagues about their clinical practice	80% (33)	100% (8)
Appreciate controversies in clinical EM	73% (30)	75% (6)
Gain critical appraisal skills in evaluating the literature	71% (29)	75% (6)
Socialize with colleagues outside of work	66% (27)	88% (7)
Improve my ability to read and understand an article	63% (26)	25% (2)
Better understand sources of bias and limitations	61% (25)	63% (5)
Translate current evidence into my clinical practice	61% (25)	86% (7)
Free food and drinks	59% (24)	25% (2)
Build good habits for my own life-long learning in EBM	56% (23)	88% (7)
Understanding research methods, study design, and statistics	49% (20)	50% (4)
Learn skills that will help me to conduct my own research	24% (10)	38% (3)

*EM*, emergency medicine; *EBM,* evidence-based medicine.

**Table 2:** Resident and faculty preference for the “traditional” vs “flipped” journal club format in terms of educational objectives and overall impressions. Total number of respondents was 33 for residents and 9 for faculty.

	Resident preference		Faculty preference		
			
	Traditional	Flipped	Traditional	Flipped	X^2^ (p-value)*
Objectives met through journal club					
Understand study design, research methods, statistics	26% (9)	74% (25)	50% (4)	50% (4)	0.76 (0.38)
Appreciate sources of bias and study limitations	26% (9)	74% (25)	38% (3)	63% (5)	0.03 (0.86)
Appreciate important controversies in clinical EM	14% (5)	86% (30)	13% (1)	88% (7)	0.19 (0.66)
Learn to select articles that might change clinical practice	12% (4)	88% (29)	13% (1)	88% (7)	0.33 (0.56)
Take valuable points from the discussion to apply to clinical practice	3% (1)	97% (33)	13% (1)	88% (7)	0.05 (0.82)
Overall impressions of journal club					
Quality of articles, topic selection	6% (2)	94% (33)	13% (1)	88% (7)	0.01 (0.92)
Social interactions with colleagues	11% (4)	89% (31)	0	100% (9)	0.17 (0.68)
Comfort with participating in discussion, asking questions	6% (2)	94% (33)	0	100% (9)	0.03 (0.86)
Overall value of time spent	6% (2)	94% (32)	0	100% (9)	0.02 (0.88)
Overall satisfaction with journal club	6% (2)	94% (33)	0	100% (9)	0.02 (0.88)

*EM*, emergency medicine.

* This chi-square test examines whether there was a difference in preference choice (traditional or flipped) for residents or faculty for each domain.
