# Supplementary material for: The Flipped Journal Club
Source: West J Emerg Med. 2017 Dec 22;19(1):23–7. doi: 10.5811/westjem.2017.11.34465 (PMC5785197; doi:10.5811/westjem.2017.11.34465)
Supplement: Supplementary file 1 [file wjem-19-23-s001.docx]

**Facilitator’s Guide for Journal Club on April 27th, 2016**

*Background Article*

**Non-invasive ventilation in community-acquired pneumonia and severe acute respiratory failure**

Carrillo, A., et al. Intensive Care Med (2012) 38: 458.

**Purpose**

- Limited evidence available on the usefulness of NIV in patients with pneumonia, as well as outcomes predicted with NIV in populations with CAP (community-acquired pneumonia) and severe ARF (acute respiratory failure)
- Study aimed to assess the characteristics and outcomes of patients with CAP and severe ARF treated with NIV while determining factors that predict failure of the technique and mortality

**Methods**

- Consecutive patients from January 1997-December 2008 in an 18-bed ICU in Murcia, Spain
  - Inclusion criteria
    - Pneumonia
  - Exclusion criteria
    - immunosuppression
    - Contraindications to NIV (agonal breathing, inability to fit mask, etc.)
  - Criteria for implementing NIV (at discretion of attending physician)
    - Moderate to severe dyspnea with RR>30 or increased work of breathing
    - PaO2/FiO2 ratio <250
- Designed to determine effectiveness of NIV
  - Defined by avoidance of endotracheal intubation and discharge from the ICU as well as remaining alive and conscious at least 24 hours after ICU discharge
- Outcomes included ICU length of stay, Hospital length of stay, ICU mortality and Hospital mortality

**Results**

- 184 patients received NIV on admission with primary diagnosis of CAP
  - 102 patients with “de novo” ARF
  - 82 patients with history of cardiac or respiratory disease
  - Overall, 116 successful with NIV, 59 failed NIV and were intubated, and 9 died without intubation after DNI order
    - NIV failure secondary to worsening ARF (39 pts), uncontrolled shock (17 pts), or NIV intolerance (3 pts)
- Variables independently associated with NIV failure
  - Worse radiologic infiltrate at 24 hours
  - Max SOFA score during NIV
  - After 1 hour of NIV: increased HR, decreased PaO2/FiO2 ratio, decreased bicarbonate
  - **De Novo ARF**
- In “de novo” ARF patients, increased duration of NIV before intubation was significantly associated with decreased hospital survival (no relationship with mortality of duration of NIV prior to intubation in patients with history of cardiac/respiratory disease)

**Limitations**

- Non-randomized trial without a control group, no definitive conclusions to be drawn
- Performed at a center with extensive NIV experience, cannot extrapolate to less trained and equipped settings

**Suggested discussion**

- Based on this article, how does NIV compare to Intubation for PNA? (hint: can’t tell from this study)
- Does a “de novo” presentation versus history of cardiac/respiratory disease play a role in your practice when deciding to use NIV in ARF?
- Should “De Novo PNA” be a contra-indication to NIV use (initial use? Continuation?)
- Should we admit patients with PNA on NIV to a non-ICU setting?

*Landmark Article*

**High-Flow Oxygen through Nasal Cannula in Acute Hypoxemic Respiratory Failure**

Frat J, et al. NEJM (2015) 372(23):2185.

**Purpose**

- This study’s aim to determine the effect of high flow oxygen on a patient with isolated hypoxic respiratory failure compared to standard oxygen therapy and non-invasive ventilation (NIV)

**Methods**

- 23 ICUs in France and Belgium, multicenter prospective study, done from 2011-2013
- Criteria to enroll in study:
  - 18 years or older
  - RR > 25
  - P/F ratio of <300 while pt on at least 10 liters for 15 mins
  - Paco2 **less than** 45
  - **No history of chronic respiratory failure**
- Randomized 1:1:1 (either high flow oxygen, standard oxygen, or NIV)
- Primary outcome was rate of intubation within 28 days*
- Secondary outcomes were mortality at 90 days, vent free days in first 28 days, and overall ICU length of stay

*Physician allowed to try NIV with high-flow oxygen or standard oxygen groups to try to prevent intubation

**Results**

- 525 met inclusion criteria (out of 2506 Hypoxic Resp Failure pts)
- Total of 310 patients enrolled and included in analysis
- On avg took 60 mins to ‘implement’ randomized therapy after enrollment in study
- **Most common cause** of acute hypoxic resp failure was community acquired PNA (64%)
- Primary outcome was intubation rate within 28 days*****
  - High flow oxygen 38%
  - Standard oxygen 47%
  - NIV 50%

*Note that these results did not reach statistical significance

- Secondary outcomes showed:
  - Significantly lower 90-day mortality in the high flow oxygen group
  - Significantly fewer vent days in high flow oxygen group

**Limitations**

- >90% of respiratory failure patients did not qualify for these study criteria
- NIV was sometimes used in the standard oxygen and high-flow groups to avoid intubation. It is unclear how often this happened
- European ICU patients (? generalizability)
- NIV Arm used HFNC b/w NIV sessions

**Suggested discussion**

- Is this applicable to patients in the ED with isolated hypoxic RF? What about patients with chronic lung disease?
- If you were to do the study again what would you change?
- What further studies are need for HFNC?
- For which patients will you consider HFNC?

*Social Media*

**Podcast 152 – High Flow Nasal Cannula – Just Blowin’ Hot Air?**

**Scott Weingart**

**June 29, 2015**

<http://emcrit.org/podcasts/hfnc/>

Definition of High Flow Nasal Cannula

-Above 15lpm, up to 60lpm

How device works

-Blowing away dead space

-Provides some CPAP up to 8cm H20 PEEP

-LPM flow is provided to nose but not transmitted to oropharynx

THRIVE Study

-Higher flows may provide increased ventilation due to increased flow down glottis

-Maintain jaw thrust to open airway structures

Miguel-Montanes Study

-HFNC better than nonrebreather mask for apneic oxygenation

[PREOXYFLOW Study](http://emcrit.org/wp-content/uploads/2015/06/PREOXY-Trial.pdf)

-HFNC not better than high flow face mask for apneic oxygenation

-Likely that device doesn’t have enough pressure to stent open airways on its own

[The FLORALI Study](http://emcrit.org/wp-content/uploads/2015/06/nejmoa1503326.pdf)

-BIPAP vs. HFNC vs. standard care

-No differences in intubation rate

-Maybe mortality reduction from HFNC is due to inappropriate intubation timing

Key points to using device:

Maintain airway patency for apneic oxygenation and possible ventilation

Close the mouth or use exhalation valve
