# Supplementary material for: The Flipped Journal Club
Source: West J Emerg Med. 2017 Dec 22;19(1):23–7. doi: 10.5811/westjem.2017.11.34465 (PMC5785197; doi:10.5811/westjem.2017.11.34465)
Supplement: Supplementary file 2 [file wjem-19-23-s002.docx]

**Appendix B.** High-Flow Nasal Cannula vs BiPAP for Acute Respiratory Failure Journal Club Summary April 2016

**Landmark Article: High-Flow Oxygen through Nasal Cannula in Acute Hypoxemic Respiratory Failure.** Frat J, et al. NEJM (2015) 372(23):2185.

Study Design: Prospective randomized multicenter study in 23 European ICUs comparing high flow oxygen, standard oxygen, and non-invasive ventilation (NIV) effect on intubation rate and mortality.

Study Limitations:

1. Patient Population: Patients with isolated hypoxic respiratory failure. Excluded patients with hypercarbia or known chronic respiratory disease. Majority had pneumonia. Limited generalizability.
2. Significant crossover:
   1. NIV Group treated with HFNC between NIV sessions
   2. Providers allowed to use NIV for patients who were failing HFNC or Standard O2
3. NIV Group used high Tidal Volumes; known to be deleterious in ARDS
4. Differences in 90 Day Mortality highlighted, though a this was a *secondary outcome*

Outcomes: Trend towards reduction in number of patients intubated at 28 days (primary outcome). Reduction in 90 Day Mortality in HFNC group (secondary outcome)

Take-Away: HFNC may be a reasonable alternative to NIV for patients with Hypoxic Respiratory Failure without Hypercarbia. Little is known about its effectiveness in patients with CHF/COPD/Asthma. Reduction in 90 Day mortality should be seen as hypothesis generating and lead to further study.

Practical Points: HFNC has promising data for reducing re-intubation, pre-oxygenation prior to RSI, and in pediatric patients. HFNC may be more tolerable than NIV for some patients. HFNC may take longer to “set-up” and is certainly less portable than NIV.

**Background Article: Non-invasive ventilation in community-acquired pneumonia and severe acute respiratory failure.** Carrillo, A., et al. Intensive Care Med (2012) 38: 458.

Study Design: Prospective observation of 184 patients in an ICU in Spain assessing patients with acute respiratory failure secondary to community-acquired pneumonia placed on non-invasive ventilation.

Take-Away:

Patients likely to “fail NIV” if, at 1 hour, there is no improvement in P/F ratio, reduction in Bicarb, higher heart rate, or higher SOFA score. Additionally, worsening radiographic appearance at 24 hours predicted failure of NIV.

Compared to patients with a history of CHF or COPD, patients with “De Novo” Respiratory Failure have a higher likelihood of failing NIV. (de novo = patients with pna, but without underlying lung disease)

Furthermore, in the “de novo RF” population, a longer duration of NIV prior to intubation was associated with *increased death*. (This was not seen in the group of patients with pre-existing heart or lung disease.) i.e. Be very cautious about letting these patients remain on NIV if they are not improving or are worsening!

**Podcast 152 – High Flow Nasal Cannula – Just Blowin’ Hot Air?**

There are theoretical advantages and drawbacks of both modalities.  BiPAP can provide greater oxygenation and ventilation support.  However, BiPAP carries risks of mucus plugging, aspiration, and impaired patient monitoring.  Clinical evidence is needed to determine which technique is better

- *Oxygenation*:  Both devices can provide close to 100% FiO2.  HFNC can provide a small and variable amount of PEEP (perhaps ~5cm, depending on the flow rate and how snugly the nasal prongs fit into the patient’s nose).  BiPAP can provide a greater amount of PEEP in a more precise fashion.
- *Work of Breathing*:  HFNC may wash out the anatomic dead space, thereby reducing the work of breathing (explained previously [here](http://www.pulmcrit.org/2014/07/high-flow-nasal-cannula-to-reduce-post.html)).  BiPAP can provide higher inspiratory pressures, and at high settings may provide the majority of the work of breathing

The trend towards harm from BiPAP is consistent with prior series showing a high failure rate of BiPAP in pneumonia.

The optimal treatment for patients with a combination of COPD and pneumonia remains unclear.  The use of BiPAP in such patients is supported by a strong track record for BiPAP in COPD exacerbations, as well as evidence from Confalonieri et al.  The optimal approach may be determined on a patient-by-patient basis, depending on the dominant disease process, cough strength, and secretion volume.

0
