# Supplementary material for: The Flipped Journal Club
Source: West J Emerg Med. 2017 Dec 22;19(1):23–7. doi: 10.5811/westjem.2017.11.34465 (PMC5785197; doi:10.5811/westjem.2017.11.34465)
Supplement: Supplementary file 3 [file wjem-19-23-s003.pdf]

Journal Club Format

1. Redesigned Journal Club Format

This is a brief, anonymous survey regarding our new journal club format that we have incorporated for the past year. We would like to know your thoughts about the new format in comparison to the traditional format we used prior to June 2015. Your input will be used to inform us on the effectiveness of the current format or to guide further changes to the journal club structure in the future.

For the remainder of the survey, "New format" refers to (1) topic and landmark article selected by residency leadership, rather than by resident group, (2) two selected articles, with incorporation of social media blog or podcast into the discussion, (3) assigned preparation for each level of training, and (4) breaking into small groups with a faculty facilitator. "Traditional format" refers to (1) assigned resident group picks the both topic and articles, and each prepares a brief presentation to be delivered to the large group, (2) each session requires reading and preparation around 3 articles, and (3) large group discussion after each article presentation, with only residents speaking for the first 5 minutes.

1. You are a

- ◯ Resident
- ◯ Attending
- ◯ Research Nurse
- ◯ Other (please specify)

[ ]

Journal Club Format

2. Attending/Research Nurse questions

2. Number of years in practice (not including residency)?

- ◯ less than 5
- ◯ 5-10
- ◯ 10-15
- ◯ more than 15

Journal Club Format

3. Resident questions

3. Year of training?

- PGY-1
- PGY-2
- PGY-3
- PGY-4
- PGY-5

4. With the traditional format, when you were designated as a "presenter," how much time did you spend reading/preparing for journal club?

- I have never been a designated presenter with the traditional format
- less than 1 hour
- 1-2 hours
- 2-3 hours
- more than 3 hours

Other (please specify)

Journal Club Format

4. Continuation of survey

5. With the **traditional** format, how much time did you spend preparing for journal club each month, on average? *(Do not include times that you were designated as a resident presenter.)*

- ◯ I'm an intern -- I never experienced the traditional format
- ◯ No time spent preparing beforehand
- ◯ less than 30 minutes
- ◯ 30 min to 1 hour
- ◯ 1 to 1.5 hours
- ◯ 1.5 to 2 hours
- ◯ 2 to 3 hours
- ◯ more than 3 hours
- ◯ Other (please specify)

6. With the **new** format, how much time do you spend preparing for journal club each month, on average? *(Do not include times that you have been a member of the resident planning group.)*

- ◯ No time spent preparing beforehand
- ◯ less than 30 minutes
- ◯ 30 min to 1 hour
- ◯ 1 to 1.5 hours
- ◯ 1.5 to 2 hours
- ◯ 2 to 3 hours
- ◯ more than 3 hours
- ◯ Other (please specify)

7. What are your personal goals/objectives for journal club? What are you looking to gain from the experience? (Check all that apply)

- [ ] Gain critical appraisal skills in evaluating the literature
- [ ] Gain an understanding of research methods, study design, and statistics
- [ ] Better understand sources of bias, limitations of published research, and learn to appropriately question new findings
- [ ] Appreciate controversies in clinical emergency medicine
- [ ] Improve my ability to read and understand an article
- [ ] Improve my knowledge of the current literature on important EM topics
- [ ] Translate current evidence into my clinical practice
- [ ] Learn from my colleagues (both residents and attendings) about their clinical practice
- [ ] Learn skills that will help me to better conduct my own research
- [ ] Promote and build good habits for my own life-long learning in evidence-based medicine
- [ ] Chance to socialize outside of work
- [ ] Free food and drinks
- [ ] Other (please specify)

8. Please rate your preference for the traditional versus the new format for journal club in regards to the following components:

	Traditional format	New format
Article and topic selection	○	○
Reading assignments, expected preparation	○	○
Social interactions with other residents and attendings	○	○
My own comfort in participating in the discussion, asking questions, offering my interpretation of the articles	○	○
Overall value of the time spent	○	○
Overall satisfaction with the journal club	○	○

Comments

9. Comparing the traditional and new formats, which format allows you to better . . .

	Traditional format	New format
Understand study design, research methods, and statistics	◯	◯
Appreciate study limitations and sources of bias	◯	◯
Appreciate the controversy surrounding important EM topics	◯	◯
Select important articles from the literature that are important for your practice	◯	◯
Take something valuable *from the articles* that you will apply to your clinical practice	◯	◯
Take something valuable *from the discussion* that you will apply to your clinical practice	◯	◯

Comments

10. Comparing the traditional and new formats, I feel like I*personally* more often . . .

	Traditional format	New format
Arrive for journal club well-prepared, having completed the assigned reading/listening	◯	◯
Contribute to the discussion	◯	◯
Ask questions	◯	◯
Have an opportunity to teach others	◯	◯

Comments

11. We are constantly looking for modifications and ways to improve journal club, but as it stands right now, **would you rather**

◯ Continue with the new format

◯ Go back to the traditional format

Comments

12. Can you suggest any ways that we can**improve** our EM Journal Club?

13. Please suggest any specific **topics or articles** that you would like to cover at Journal Club over the next year.
